# High-Accuracy Globally Consistent Surface Reconstruction Using Fringe Projection Profilometry

**DOI:** 10.3390/s19030668

**Published:** 2019-02-06

**Authors:** Xu Cheng, Xingjian Liu, Zhongwei Li, Kai Zhong, Liya Han, Wantao He, Wanbing Gan, Guoqing Xi, Congjun Wang, Yusheng Shi

**Affiliations:** 1 State Key Laboratory of Material Processing and Die & Mould Technology, Huazhong University of Science and Technology, Wuhan 430074, China; xu_cheng@hust.edu.cn (X.C.); xingjianliu@hust.edu.cn (X.L.); hly1993@hust.edu.cn (L.H.); walden@263.net (C.W.); shiyusheng@hust.edu.cn (Y.S.); 2 School of Mechanical Engineering, Heilongjiang University of Science and Technology, Harbin 150022, China; wantaohe@hust.edu.cn; 3 Hubei Tri-Ring Forging Co., Ltd, Gucheng 441700, China; wbgan1@163.com (W.G.); gqxi11@163.com (G.X.)

**Keywords:** quality control, fringe projection profilometry, depth image registration, 3D reconstruction

## Abstract

This paper presents a high-accuracy method for globally consistent surface reconstruction using a single fringe projection profilometry (FPP) sensor. To solve the accumulated sensor pose estimation error problem encountered in a long scanning trajectory, we first present a novel 3D registration method which fuses both dense geometric and curvature consistency constraints to improve the accuracy of relative sensor pose estimation. Then we perform global sensor pose optimization by modeling the surface consistency information as a pre-computed covariance matrix and formulating the multi-view point cloud registration problem in a pose graph optimization framework. Experiments on reconstructing a 1300 mm × 400 mm workpiece with a FPP sensor is performed, verifying that our method can substantially reduce the accumulated error and achieve industrial-level surface model reconstruction without any external positional assistance but only using a single FPP sensor.

## 1. Introduction

Fringe projection profilometry provides a convenient way to measure dense and accurate three dimensional (3D) surface point cloud of target objects. It plays an increasingly important role in various fields such as industrial quality inspection, prototyping, culture heritage preservation and movie industry [1,2,3,4,5]. Owing to the limited field of view (FOV) and object self-occlusion, 3D point cloud obtained from a single viewpoint only contains partial surface shape data. To reconstruct complete surface models, 3D measurements from multiple viewpoints are deserved to cover the whole object, and their sensor poses need to be precisely tracked to further transform these partial surface point clouds into a global coordinate system [6,7,8,9].

Existing sensor pose tracking solutions are mostly based on external assistance methods, such as attaching artificial markers or using external positional equipment such as a laser tracker or optical coordinate measuring machines (CMMs) [10], their usage flexibility is inherently limited. Alternatively, sensor poses can also be directly estimated by using 3D registration techniques [11,12,13] to compute the relative pose between sequential two measurements. However, sensor pose estimation drifts inevitably exist due to 3D registration inaccuracy. Small sensor pose estimation error which may seem negligible on a local scale, can drastically accumulate along a long scanning trajectory [12,14]. The accumulated error directly leads to surface point clouds inconsistency between the first and last scans and finally breaks the reconstruction result.

Different optimization methods have been adopted to solve the accumulated error problem. Among them, bundle adjustment (BA) is one of the most well-known approaches that performs global optimization by minimizing the reprojection error across different frames. Specifically, BA is conducted by firstly identifying the same visual feature points appearing in multiple frames, and then adjusting the estimated 3D locations of feature points together with the camera poses [7,9]. Nevertheless, BA only optimizes sparse 3D feature points and camera poses, thus it does not guarantee local shape consistency of the reconstructed 3D models [14]. Besides, visual feature detection is the prerequisite for BA optimization, it cannot be fulfilled when the color image is not valid or the target object surface is textureless (e.g., industrial parts).

Instead of optimizing the accumulated error to solve surface inconsistency, Zhou et al. [15] and Whelan el al. [16] chose to deform inconsistency local point clouds together using non-rigid 3D registration techniques, consumer RGB-D sensors are taken as the depth input in their works. Shape deformation provides a simple yet useful approach to obtain globally consistent models, especially in some applications such as indoor reconstruction [12] where surface consistency instead of the accuracy is of the most importance. However, shape deformation is not desired in our problem, because it directly ruins the surface measurement accuracy. Furthermore, since FPP sensor provides high-accuracy surface point cloud measurements, theoretically when sufficient accurate sensor poses are recovered, the individual local 3D point clouds should be able to integrate into a globally consistent model using only rigid transformations.

Differently, Cao et al. [17] and Yue et al. [18] optimized the accumulated error by first identifying the loop closures formed through successful 3D registration between each current frame and other earlier frames, and then performing a pose graph optimization [19] to reduce the sensor poses drifts. However, in their works the loop closures are identifying either by manually checking the 3D point cloud overlapping ratio [17], or by using the measurement system setup information [18], which prevents their further usage in a practical 3D scanning system. Moreover, the pose graph optimization in [17,18] only optimized the inconsistency between two associated sensor poses and their relative pose constraint; it ignores important surface consistency information in the 3D registration process [6].

According to the above analysis, the key to accurate surface reconstruction lies in the reduction of accumulated sensor pose estimation error. In this paper, we present a flexible and accurate method for high-accuracy globally consistent surface reconstruction using a single FPP sensor. The accumulated error problem is addressed from two aspects: (1) observing the underlying principle that surface curvature remains invariant against measurement viewpoint changes, a novel 3D registration method is proposed which fuses both dense geometric and curvature consistency constraints to joint optimize the relative sensor pose estimation. The introduction of curvature consistency constraint implicitly pays attention to high-curvature surfaces, which helps to generate more accurate 3D registration results [20]. (2) We utilize 6-DOF pose distances for adaptive keyframe determination, and use a two-step checking scheme for automatic loop closure detection. By modelling the surface inconsistency information as a pre-computed covariance matrix and formulating the multi-view point cloud registration problem in a pose graph optimization framework, the accumulated error can be effectively reduced to obtain the final accurate sensor pose estimations.

The effectiveness of our proposed method is demonstrated by reconstructing a 1300 mm × 400 mm workpiece with a FPP sensor. Results show that the proposed method substantially reduced the accumulated error, making the sensor pose estimation accuracy match the measurement accuracy well. Our method shows the ability to accomplish industrial-level surface model reconstruction without any external positional assistance but only using a single FPP sensor.

## 2. Measurement Principle

In our FPP sensor, a series of sinusoidal fringes along the horizontal axes of projector image frame with constant phase shifting are projected onto a target object, and two cameras capture the distorted fringe images synchronously. The captured images can be expressed as:

(1) $$I_{i}\left(x,y\right)=A_{i}\left(x,y\right)+B_{i}\left(x,y\right)\cos\left(\phi \left(x,y\right)+\delta_{i}\right),\;i=1,2,3,\ldots ,n$$

where (x,y) is the pixel coordinates and is omitted in the following expression, $I_{i}$ denotes the recorded intensity, $A_{i}$ indicates the average intensity, $B_{i}$ represents the modulation intensity, $\delta_{i}$ is the constant phase-shift, *n* is the phase shift number, and ϕ is the desired phase information. By solving Equation (1), the phase value ϕ can be obtained according to:

(2) $$\phi =-\arctan(\sum_{i=1}^{n}I_{i}\sin\left(\delta_{i}\right)/\sum_{i=1}^{n}I_{i}\cos\left(\delta_{i}\right)).$$

**Figure 1:**
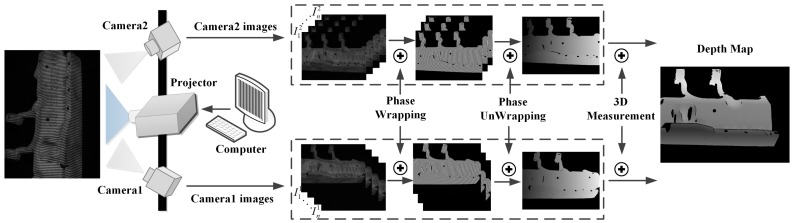
The 3D measurement principle of fringe projection profilometry (FPP sensor).

The arctangent function in Equation (2) will result in a phase value within the range of [−π,π] with 2π discontinuities. In our sensor, multi-frequency heterodyne technology is adopted to construct the continuous phase map [21], so that the correspondence between two camera views can be established unambiguously. Finally, the 3D result can be obtained according to the pre-calibrated camera intrinsic and external parameters. The measurement principle of the FPP sensor is shown in the Figure 1 below.

## 3. Relative Sensor Pose Estimation

The relative sensor pose estimation between sequential two measurements (also called as frames in the following) is the basis to obtain the initial global sensor pose estimation of each measurement. In this section, we will introduce the proposed method which estimates the relative sensor pose (a rigid transformation) by 3D registering two depth maps to jointly optimize the dense geometric and curvature inconsistency errors. The whole process is conducted by first computing the curvature map of each depth map, and then iteratively performing data association and error minimization steps.

### 3.1. Curvature Map Estimation

Similarly to depth map (also called as depth image), curvature map is a 2D image in which the value of each pixel is the surface curvature value instead of the depth value. Specifically, for each pixel $x={\left(u,v\right)}^{⊺}$ in the depth map with valid depth z(x), its corresponding 3D point coordinate p(x) can be computed using the inverse of projection function Π(·) as:

(3) $$\begin{array}{cc} p(x) & =\Pi^{-1}\left(x,z\left(x\right)\right)\\ & =z\left(x\right){\left(\frac{u-c_{x}}{f_{x}},\frac{v-c_{y}}{f_{y}},1\right)}^{⊺}, \end{array}$$

where $f_{x}$, $f_{y}$ are the focal lengths and $c_{x}$, $c_{y}$ are the principle point, respectively. The mean curvature of each point on the surface is represented using a surface variation notion in [22]. Hence, the surface curvature value κ(x) at pixel x is estimated by taking the eigen-analysis of the covariance matrix of the local neighbor points of point p(x). The covariance matrix is defined as:

(4) $$C\left(x\right)=\sum_{i}^{k}\left(p_{i}-\bar{p}\right){\left(p_{i}-\bar{p}\right)}^{⊺},\;\bar{p}=\frac{1}{k}\sum_{i}^{k}p_{i},$$

where $p_{i}$ is one of the nearest neighbor points of p(x). Then κ(x) can be computed as:

(5) $$\kappa \left(x\right)=\frac{\lambda_{0}}{\lambda_{0}+\lambda_{1}+\lambda_{2}},$$

where $\lambda_{0}\leq \lambda_{1}\leq \lambda_{2}$ are the eigenvalues of the covariance matrix C(x).

To speed up the nearest neighbor search, we take advantage of the organized point cloud structure embedded in the depth map, only taking adjacent pixels as candidate neighbors. Meanwhile, the geometric continuity constraints are also considered to filter the potential depth gaps by specifying a maximum allowed distance. Pixel $x_{i}$ is the nearest neighbor of pixel x, only when it satisfies $∥x-x_{i}∥\leq \sigma_{1}$, and $∥p\left(x\right)-p\left(x_{i}\right)∥\leq \sigma_{2}$, where $\sigma_{1}$ and $\sigma_{2}$ represent the pixel and point nearest neighbor distance threshold, respectively. In this paper, we set $\sigma_{1}=3$ and $\sigma_{2}=1.1$ mm (with average point cloud density as 0.275 mm) to allow approximate 30 nearest neighbor points for curvature value estimation.

**Figure 2:**
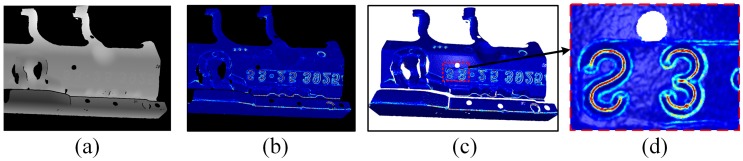
(**a**) A depth map acquired with the FPP sensor, (**b**) Its corresponding curvature map estimated using our method, (**c**) The rendered 3D point cloud with its color mapped from the curvature map, (**d**) Local details of curvature information at local point cloud surface.

Figure 2a shows a depth map measured with the FPP sensor, Figure 2b shows the estimated curvature map using our method. Figure 2c is the corresponding 3D point cloud whose color is mapped from the curvature map, and the local detail is displayed in Figure 2d. It can be seen that the estimated curvature map exhibits high consistency with the point cloud surface variation. Furthermore, by carefully handling the discontinuous boundary case, the curvature values at boundary points can also be robustly estimated, as shown in Figure 2d.

### 3.2. Data Association

Data association is to identify the corresponding points between two sequential frames, the correspondence set is then fed to the optimization process to find the optimal relative sensor pose estimation. Assuming small camera motion between sequential frames, the projective data association algorithm [12] is conducted to produce the point correspondences set. Given the relative sensor pose estimation $T_{i-1,i}$ between current frame $f_{i}$ and its previous frame $f_{i-1}$, then for each pixel x with valid depth in $f_{i}$, we first transform its corresponding 3D point p(x) into the local coordinate system of previous frame $f_{i-1}$ as $T_{i-1,i}p\left(x\right)={\left(x,y,z\right)}^{⊺}$. Then the corresponding pixel of x in frame $f_{i-1}$ can be computed with perspective projection:

(6) $$\begin{array}{cc} \bar{x} & =\Pi (T_{i-1,i}p\left(x\right))\\ & =\frac{1}{z}KT_{i-1,i}p\left(x\right)\\ & ={\left(f_{x}\frac{x}{z}+c_{x},f_{y}\frac{y}{z}+c_{y}\right)}^{⊺}, \end{array}$$

where K is the camera intrinsic matrix. Note that for simplicity of notation, we omit the conversions between vectors and its homogeneous vectors throughout this paper.

With the projective data association, multiple pixels in source depth image $f_{i}$ may correspond to a common pixel in target depth image $f_{i-1}$. To solve the many-to-one problem, the *z*-buffer technique is adopted, for each pixel in target depth map $f_{i-1}$ we only keep the corresponding pixel in source depth map $f_{i}$ with minimum depth. All corresponding points pairs together construct the corresponding set $K_{i-1,i}=\left\{\left(x,\bar{x}\right)\right\}$ between frame $f_{i}$ and $f_{i-1}$.

### 3.3. Minimization

The relative sensor pose optimization function $E_{reg}$ is defined as:

(7) $$E_{reg}=E_{geo}+\lambda E_{cur},$$

where $E_{geo}$ denotes the geometric inconsistency error, $E_{cur}$ denotes the curvature inconsistency error, λ is the weight of the curvature inconsistency error.

The geometric error is defined as the point-to-plane error [11] between current and previous frames:

(8) $$E_{geo}=\sum_{\left(x,\bar{x}\right)\in K_{i-1,i}}{∥\left(\exp\left(\hat{\xi }\right)T_{i-1,i}p_{i}\left(x\right)-p_{i-1}\left(\bar{x}\right)\right)\cdot n_{i-1}\left(\bar{x}\right)∥}^{2},$$

in which $\left(x,\bar{x}\right)$ is one corresponding pixels pair in the corresponding set $K_{i-1,i}$, $p_{i}\left(x\right)$ is the local 3D point in the current frame $f_{i}$, $p_{i-1}\left(\bar{x}\right)$ and $n_{i-1}\left(\bar{x}\right)$ are the corresponding 3D point and normal, respectively. $T_{i-1,i}$ is the current estimation of the relative sensor pose between the two frames. $\exp\left(\hat{\xi }\right)\in \mathrm{SE}\left(3\right)$ is the incremental transformation to be estimated in each iteration, in which $\xi ={\left(\omega ,t\right)}^{⊺}={\left(\alpha ,\beta ,\gamma ,t_{x},t_{y},t_{z}\right)}^{⊺}\in R^{6}$.

The curvature inconsistency error $E_{cur}$ is defined as the curvature value inconsistency between the warped curvature map of current frame $f_{i}$ and the curvature map of previous frame $f_{i-1}$:

(9) $$\begin{array}{cc} E_{cur} & =\sum_{\left(x,\bar{x}\right)\in K_{i-1,i}}{∥\kappa_{i}\left(x\right)-\kappa_{i-1}\left(\bar{x}\right)∥}^{2}\\ & =\sum_{\left(x,\bar{x}\right)\in K_{i-1,i}}∥\kappa_{i}\left(x\right)-\kappa_{i-1}{(\Pi \left(\exp\left(\hat{\xi }\right)T_{i-1,i}p_{i}\left(x\right)\right)∥}^{2}, \end{array}$$

where $\kappa_{i}\left(x\right)$ is the curvature value at pixel x of the current frame, $\kappa_{i-1}\left(\bar{x}\right)$ is the curvature value at pixel $\bar{x}$ of the previous frame.

Assuming the incremental pose transformation $\exp(\hat{\xi })$ to optimize at each iteration is small, it can be linearized as $\exp\left(\hat{\xi }\right)\approx I+\hat{\xi }$, where $\hat{\xi }\in \mathrm{se}\left(3\right)$ is the corresponding Lie algebra element:

(10) $$\hat{\xi }=\left[\begin{array}{cc} {\left[\omega \right]}_{\times } & t\\ 0^{⊺} & 0 \end{array}\right]=\left[\begin{array}{cccc} 0 & -\gamma & \beta & t_{x}\\ \gamma & 0 & -\alpha & t_{y}\\ -\beta & \alpha & 0 & t_{z}\\ 0 & 0 & 0 & 0 \end{array}\right],$$

the ${\left[\cdot \right]}_{\times }:R^{3}\to \mathrm{so}\left(3\right)$ is a linear skew-symmetric operator (see [23] for details).

With this linearization and simple notation ${\dot{p}}_{i-1}\left(x\right)=T_{i-1,i}p_{i}\left(x\right)$, the error term $E_{geo}$ becomes:

(11) $$\begin{array}{cc} E_{geo} & \approx \sum_{\left(x,\bar{x}\right)\in K_{i-1,i}}{∥\left(\left(I+\hat{\xi }\right){\dot{p}}_{i-1}\left(x\right)-p_{i-1}\left(\bar{x}\right)\right)\cdot n_{i-1}\left(\bar{x}\right)∥}^{2}\\ & =\sum_{\left(x,\bar{x}\right)\in K_{i-1,i}}{∥{\left[\begin{array}{c} p_{i-1}\left(x\right)\times n_{i-1}\left(\bar{x}\right)\\ n_{i-1}\left(\bar{x}\right) \end{array}\right]}^{⊺}\xi +\left({\dot{p}}_{i-1}\left(x\right)-p_{i-1}\left(\bar{x}\right)\right)\cdot n_{i-1}\left(\bar{x}\right)∥}^{2}\\ & =∥J_{geo}\xi +r_{geo}{∥}^{2}, \end{array}$$

where $J_{geo}$ is the Jacobian matrix and $r_{geo}$ is the residual vector. Similarly, the error term $E_{cur}$ becomes:

(12) $$\begin{array}{cc} E_{cur} & \approx \sum_{\left(x,\bar{x}\right)\in K_{i-1,i}}{∥\kappa_{i}\left(x\right)-\kappa_{i-1}\left(\Pi \left(\left(I+\hat{\xi }\right){\dot{p}}_{i-1}\left(x\right)\right)\right)∥}^{2}\\ & =\sum_{\left(x,\bar{x}\right)\in K_{i-1,i}}{∥\kappa_{i}\left(x\right)-\kappa_{i-1}\left(\frac{1}{z}K\left(I+\hat{\xi }\right){\dot{p}}_{i-1}\left(x\right)\right)∥}^{2}\\ & \approx \sum_{\left(x,\bar{x}\right)\in K_{i-1,i}}{∥-\frac{\partial \kappa_{i-1}\left(\bar{x}\right)}{\partial \bar{x}}\frac{\partial \bar{x}}{\partial \hat{\xi }{\dot{p}}_{i-1}\left(x\right)}\frac{\partial \hat{\xi }{\dot{p}}_{i-1}\left(x\right)}{\partial \xi }\xi +\kappa_{i}\left(x\right)-\kappa_{i-1}\left(\frac{1}{z}K{\dot{p}}_{i-1}\left(x\right)\right)∥}^{2}\\ & =∥J_{cur}\xi +r_{cur}{∥}^{2}. \end{array}$$

With the above linearization, minimization of Equation (7) allows to solve the following linear system:

(13) $$\left(J_{geo}^{⊺}J_{geo}+\lambda J_{cur}^{⊺}J_{cur}\right)\xi =-\left(J_{geo}^{⊺}r_{geo}+\lambda J_{cur}^{⊺}r_{cur}\right).$$

In each iteration, we compute Jacobian $J_{geo}$, $J_{cur}$ and residual $r_{geo}$, $r_{cur}$ at current relative sensor pose estimation $T_{i-1,i}$, and solve the linear system in Equation (13) to find the ξ that best satisfies the geometric and curvature consistency constraint. Then the relative pose $T_{i-1,i}$ is updated to $\exp\left(\hat{\xi }\right)T_{i-1,i}$, and taken as the initialization for the next iteration.

When the optimization converges, the $T_{i-1,i}$ is taken as the final relative sensor pose estimation between two frames. We fix the sensor pose of the first frame $f_{1}$ as $T_{1}=I$ and regard it as the world coordinate system. Then the initial global sensor pose of frame $f_{i}$ is computed as $T_{i}=T_{i-1}T_{i-1,i}$.

**Figure 3:**
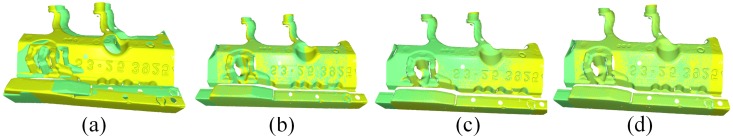
(**a**) Initial relative pose between source (green) and target (yellow) point cloud, (**b**) Registration result by only minimizing geometric error in Equation (8), (**c**) Point-to-plane ICP performed on 3D point cloud with a max distance threshold to eliminate outliers, (**d**) Minimizing both of the geometric error and curvature error as proposed in this paper.

Figure 3 shows the 3D registration results comparison between the proposed method and two other methods. The sensor pose estimation accuracy is directly reflected in the surface shape consistency of two registered point clouds. When independently visual inspecting each registration result, each method seems to converge to a correct result. However, when comparing the registration results between Figure 3b–d, it is not hard to see that the relative sensor pose estimation accuracy of our method outperforms the other two methods.

**Figure 4:**
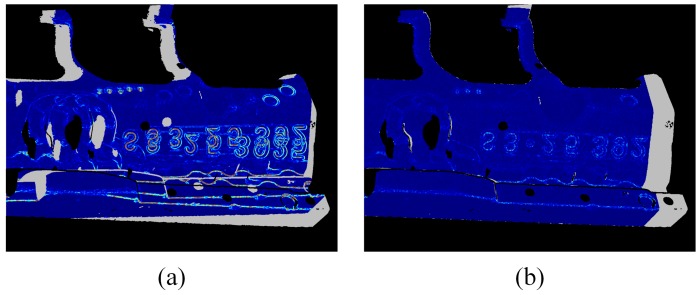
Curvature difference map (**a**) Before registration, (**b**) After registration.

Figure 4a,b represents the curvature value difference map between source and target point cloud before and after the 3D registration, respectively. The curvature difference map is built on the target frame $f_{i-1}$, correspondences are built using the above data association method. Gray pixels indicate that no correspondence is built for these pixels. It can be seen that the curvature value difference from Figure 4a,b decreases dramatically over the whole map, which demonstrates the significance of introducing curvature map consistency into the 3D registration constraints.

## 4. Global Sensor Pose Optimization

Though fusing curvature consistency information improves the accuracy of the estimated relative sensor poses, the global sensor pose drift will inevitably accumulate during a long scanning process. To reduce the accumulated error and obtain globally consistent 3D models, successful relative pose estimation to much earlier frames (also called as building *loop closure*) is deserved. In this section, we will first introduce how to automatically build a series of loop closures with the proposed adaptive keyframe selection and the two-step checking method. We will then introduce our method which performs multi-view point cloud registration in a pose graph optimization framework [19].

### 4.1. Keyframe Selection

Detecting loop closure for every new-income measurement is not optimal; it will greatly increase the computation cost after a long time scanning. Therefore, we only detect loop closure for selected keyframes. We utilize 6-DOF (degree of freedom) pose distance metrics to determine when to add a new keyframe for further loop closure detection. For each new input frame $f_{j}$, we evaluate the relative pose distances between it and the last added keyframe $f_{i-1}^{k}$. In which, the rotation distance is measured as the rotation angle using the Rodrigues’ formula:

(14) $$d\left(R_{j},R_{i-1}^{k}\right)=\left|\arccos\left(\frac{trace(R_{j}^{⊺}R_{i-1}^{k})-1}{2}\right)\right|.$$

The translation distance is computed as:

(15) $$d\left(t_{j},t_{i-1}^{k}\right)=∥t_{j}-t_{i-1}^{k}∥,$$

**Figure 5:**
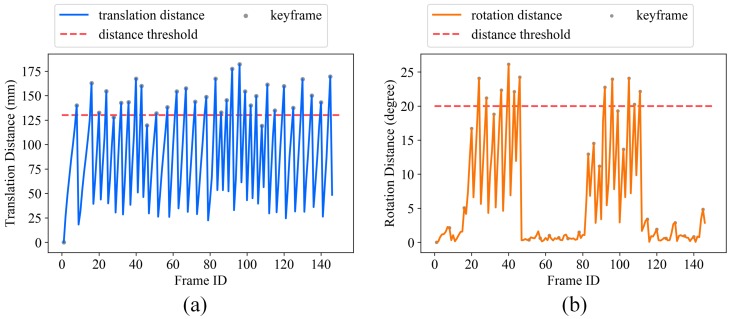
(**a**,**b**) show the translation and rotation distance between each frame with its previous keyframe, respectively.

If either the rotation or translation distance exceeds its corresponding threshold $\sigma_{R}$ or $\sigma_{t}$, the current frame $f_{j}$ is marked as a new keyframe $f_{i}^{k}$. We set $\sigma_{R}=20^{\circ }$, $\sigma_{t}=130$ mm in our paper. Figure 5 shows the keyframe selection results using the total 146 depth maps acquired with our FPP sensor (see Section 5). Gray points identify the 34 selected keyframes out of a total 146 depth maps.

### 4.2. Loop Closure Detection

For each new added keyframe $f_{i}^{k}$, we use a two-step checking scheme to detect whether it forms correct loop closures with previous keyframes. If two keyframes construct a loop closure, then they must fulfil: (1) the overlapping area between two point clouds is enough, (2) the mean absolute error (MAE) between them is small.

**Figure 6:**
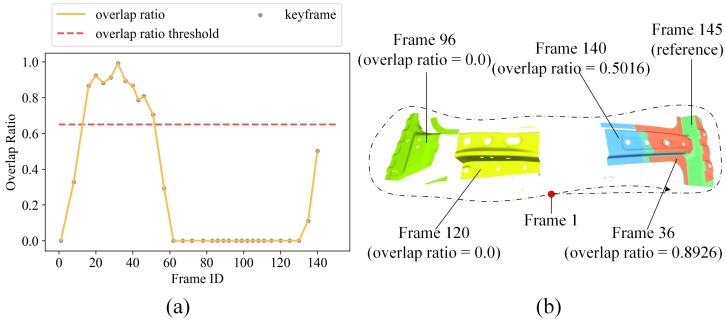
(**a**) The computed overlapping ratios between frame 145 with all its previous keyframes and (**b**) Frame 36, 96, 120, 140 and the reference frame 145.

The overlapping area ratio is crucial for arbitrary two frames with loop closures, as small overlapping area ratios are prone to correspond to non-loop-closure connection. In this paper, we propose to use the projective association algorithm to efficiently compute the overlapping area ratio between two keyframes. When a new keyframe $f_{i}^{k}$ arrives, we compute its depth valid map $V_{i}^{k}$ for each pixel. $V_{i}^{k}\left(x\right)=1$ for each pixel where its depth is valid, and $V_{i}\left(x\right)=0$ when depth is not valid. Then for a pair of keyframes $f_{i}^{k}$ and $f_{j}^{k}$, we obtain the correspondence set $K_{i,j}^{k}=\left\{\left(x,\bar{x}\right)\right\}$ using the data association method in Section 3.2. Note that, the relative sensor pose between $f_{i}^{k}$ and $f_{j}^{k}$ is computed as $T_{i,j}^{k}={T_{i}^{k}}^{-1}T_{j}^{k}$ here. A correspondence pair $\left(x,\bar{x}\right)$ is identified as overlapped when $V_{j}^{k}\left(\bar{x}\right)=1$. We collect all overlapped point pairs, the overlapping ratio is computed as $\tau_{o}=N/M$, where *N* is the overlapped points number, *M* is the total number of points with valid depth. If the overlapping ratio $\tau_{o}$ is larger than the threshold $\sigma_{o}$, we mark keyframe $f_{i}^{k}$ and $f_{j}^{k}$ as a candidate loop closure. Figure 6a shows the overlapping ratios between the 34th keyframe (frame 145) with all its previous keyframes, we set the overlapping ratio threshold $\sigma_{o}=0.65$ in this paper. We select frame 36, 96, 120 and 140 to visualize the correctness of our proposed method as shown in Figure 6b, dotted line sketches the scanning path.

We then check the dense geometric consistency to further validate the correctness of these candidate loop closures. A candidate loop closure $\left(f_{i}^{k},f_{j}^{k}\right)$ is considered as reliable only if the MAE of the correspondence points between two frames is below a threshold $\sigma_{r}$:

(16) $$\frac{1}{|K_{i,j}|}\sum_{\left(x,\bar{x}\right)\in K_{i,j}}∥T_{i,j}^{k}p\left(x\right)-p\left(\bar{x}\right)∥<\sigma_{r}.$$

If the two-step checks all passed, the two frames are further registered together to construct a loop closure.

### 4.3. Graph Based Sensor Pose Optimization

Removing the accumulated error to get globally consistent model needs to eliminate the surface inconsistencies across all associated point clouds. We define the surface inconsistency as a error term $F_{i,j}$ in terms of the dense geometric registration error between frame $f_{i}$ and $f_{j}$, as:

(17) $$\begin{array}{cc} F_{i,j} & =\sum_{p_{i},p_{j}}{∥\left(T_{j}p_{j}-T_{i}p_{i}\right)∥}^{2}\\ & =\sum_{p_{i},p_{j}}{∥T_{i}^{-1}T_{j}p_{j}-p_{i}∥}^{2}\\ & \approx \sum_{p_{i},p_{j}}{∥T_{i}^{-1}T_{j}T_{j,i}p_{i}-p_{i}∥}^{2}. \end{array}$$

Note that $T_{i}$, $T_{j}$ is obtained through the relative sensor pose estimation in Section 3.3, $T_{j,i}$ is obtained through the loop closure detection in Section 4.2. Inconsistency exists between $T_{j,i}$ and $T_{i}$, $T_{j}$ due to the accumulated error. Line (17) holds by restricting the corresponding points $\left(p_{i},p_{j}\right)$ must fulfil $∥T_{j,i}p_{i}-p_{j}∥<\epsilon$, we set ϵ=1.0 mm in this paper.

Then by approximating $T_{i}^{-1}T_{j}T_{j,i}=I+{\hat{\xi }}_{i,j}$, Equation (17) can be written as:

(18) $$\begin{array}{cc} F_{i,j} & \approx \sum_{p_{i},p_{j}}{∥{\hat{\xi }}_{i,j}p_{i}∥}^{2}\\ & =\sum_{p_{i},p_{j}}{∥\left[\begin{array}{cc} -{\left[p_{i}\right]}_{\times } & I \end{array}\right]\xi_{i,j}∥}^{2}, \end{array}$$

in which $\xi_{i,j}$ actually measures the inconsistency between sensor pose $T_{i}$, $T_{j}$ and their relative pose constraint $T_{i,j}$. Define $G_{i}=\left[\begin{array}{cc} -{\left[p_{i}\right]}_{\times } & I \end{array}\right]$, we obtain:

(19) $$\begin{array}{cc} F_{i,j} & \approx \sum_{p_{i},p_{j}}{∥G_{i}\xi_{i,j}∥}^{2}\\ & =\xi_{i,j}^{⊺}\sum_{p_{i},p_{j}}G_{i}^{⊺}G_{i}\xi_{i,j}\\ & =\xi_{i,j}^{⊺}\Omega_{i,j}\xi_{i,j}. \end{array}$$

Equation (19) shows the surface inconsistency term $F_{i,j}$ can be represented with the sensor pose inconsistency term $\xi_{i,j}$ and a covariance matrix $\Omega_{i,j}=\sum_{p_{i},p_{j}}G_{i}^{⊺}G_{i}$, it is constant and can be pre-computed for each term during the 3D registration process.

Let C be the set of indices for which a connection between two sensor poses exists, then the multi-view point cloud registration problem can be formulated as:

(20) $$\begin{array}{cc} F & =\sum_{(i,j)\in C}F_{i,j}\\ & =\sum_{(i,j)\in C}\xi_{i,j}^{⊺}\Omega_{i,j}\xi_{i,j}. \end{array}$$

**Figure 7:**
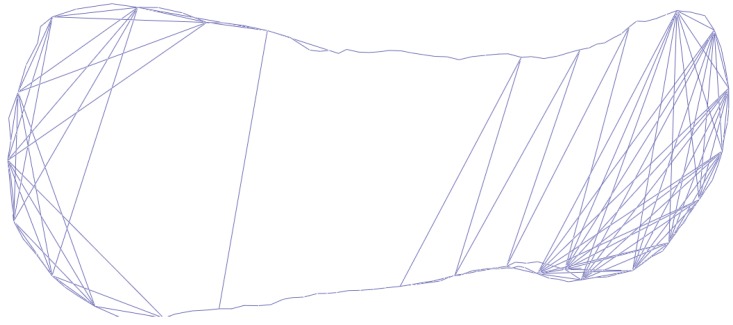
It shows a pose graph consists of 146 pose vertices, 229 edges (84 loop closure edges inside).

This exactly defines a pose graph optimization, which can be directly solved using the *g2o* library [19]. Figure 7 shows the pose graph constructed with our method. Vertices represent the 6-DoF sensor poses, edges represent the constraints between sensor poses. The pose graph is visualized with the *g2o viewer* software.

## 5. Experiment

**Figure 8:**
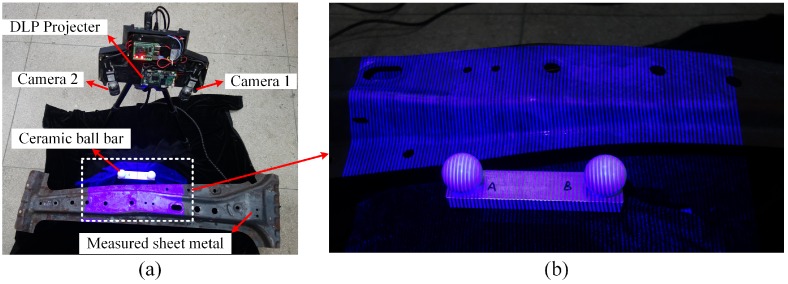
(**a**) The measurement scene, (**b**) Sinusoidal fringe pattern projected onto the measured object.

In the experiment, a FPP sensor is constructed using (1) a Texas Instruments LighterCrafter4500 board (Texas Instruments, Dallas, TX, USA) for fringe patterns projection, (2) two Basler acA1300-30gm cameras (Basler AG, Ahrensburg, Germany) simultaneously capturing the modulated images with pixel resolution of 1296 × 966. The proposed method is validated by scanning a 1300 mm × 400 mm sheet metal using the FPP sensor as shown in Figure 8, the 3D measurement and model reconstruction are conducted on a desktop PC with a 3.3 GHz Intel Xeon CPU and 16 GB RAM. By moving the FPP sensor around, complete scan of the sheet metal with totally 146 frames (depth maps) acquired is accomplished.

To test and verify the accuracy and effectiveness of the proposed relative sensor pose estimation method and the global optimization method, a ceramic ball bar is placed beside the measured sheet metal. The reconstruction accuracy can then be well examined by qualitatively observing the surface consistency and quantitatively analyzing the size fitting results of the reconstructed ceramic ball bar.

### 5.1. Relative Sensor Pose Estimation Accuracy

**Figure 9:**
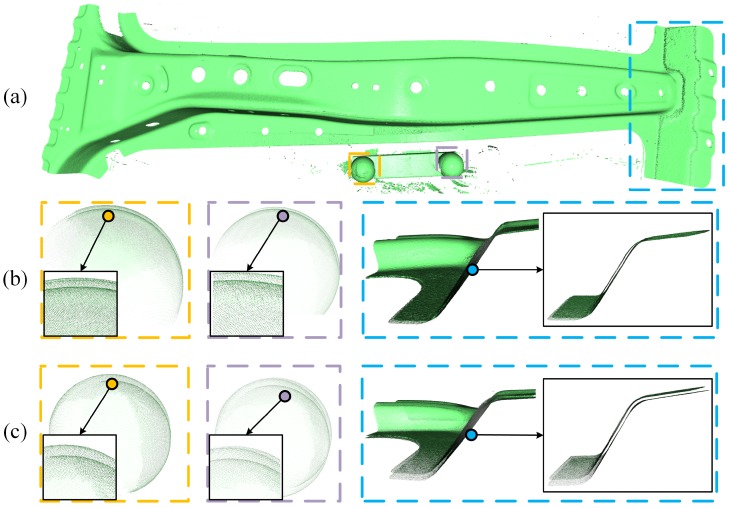
(**a**) The reconstructed surface and (**b**) Its local details using both geometric and curvature consistency constraints, (**c**) The corresponding local details using only geometric consistency constraints (its complete surface model not displayed here).

The accuracy of our proposed relative sensor pose estimation method is tested first. The sensor pose of each frame relative to the world coordinate system (frame 1) is separately estimated by (1) jointly optimizing the geometric and curvature consistency constraints (our method), (2) only optimizing the geometric consistency constraint for comparison. With the estimated sensor poses, 3D point cloud of each frame is transformed to the world coordinate system and further voxel downsampled to a unified 3D point cloud. Figure 9a shows the reconstructed surface of sheet metal with our method, it shows that the overall shape of our reconstruction result matches the actual sheet metal shape well. The point clouds are rendered with *Open3D* library [24].

On the other side, sensor pose estimation error inevitably accumulated in the reconstruction process, which leads to obvious surface shape artifacts, as shown in Figure 9b,c. In which, Figure 9b shows the local surface inconsistency at 3 difference places using our method, Figure 9c shows the corresponding results using only geometric consistency constraints. With this comparison, it is not hard to see that introducing the curvature consistency constraint effectively improves the sensor pose estimation accuracy, which provides a good foundation for further global optimization.

### 5.2. Global Sensor Pose Optimization Accuracy

**Figure 10:**
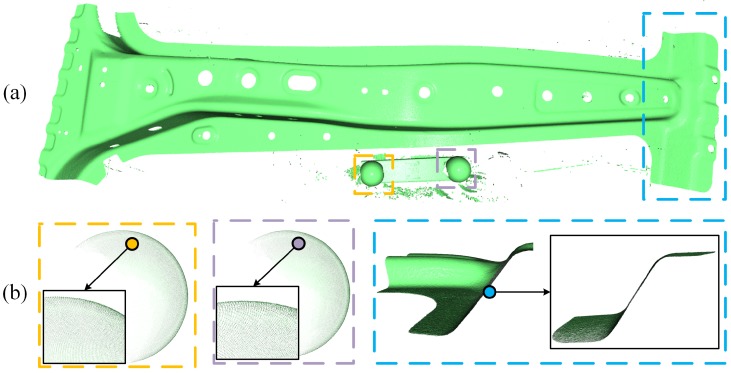
(**a**) The reconstructed surface after global optimization, (**b**) Its local details.

Based on the sensor pose estimation results above, the global optimization is performed by (1) keyframe selection, (2) loop closure detection and (3) pose graph optimization. Then the globally optimized reconstruction result is obtained with the optimized sensor poses. Figure 10a,b show the optimized surface model and its local details, respectively. With the global model optimization, we obtained globally consistent surface model, surface inconsistencies due to the accumulated error are well optimized as shown in Figure 10b.

**Figure 11:**
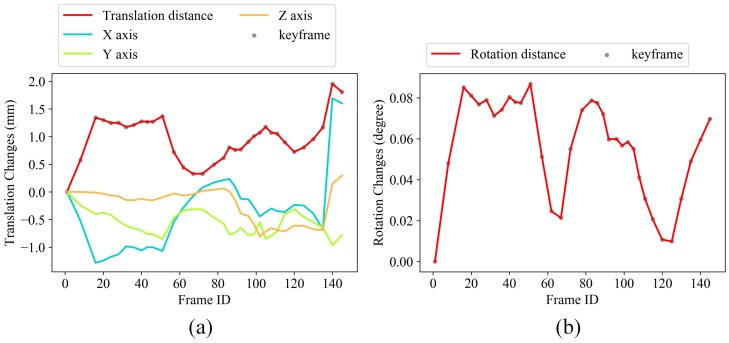
(**a**) Relative translation and (**b**) Rotation changes of each keyframe pose.

To further quantitatively analyze the accuracy improvement with the global optimization, we computed the relative translation and rotation changes of each keyframe pose before and after global optimization, as shown in Figure 11, the optimized poses are taken as the reference values here. It demonstrates that even very small translation estimation inaccuracy (less than 2.0 mm) and rotation estimation inaccuracy (less than $0.10^{\circ }$) in the reconstruction range of 1300 mm × 400 mm, are enough to cause obvious surface inconsistency (as shown in Figure 9b), and lead to reconstruction results that are unusable for high-accuracy dimensional inspection.

Meanwhile, the absolute accuracy of the reconstructed surface model can be directly and precisely tested by comparing (1) diameter fitting values of two spheres, (2) standard deviation values of Euclidean distances between sphere surface 3D points and the fitted sphere surface, (3) Euclidean distance between two sphere centers. The comparison is made between the not-optimized model, globally-optimized model and the ground truth. The ground-truth is obtained with the fitting values of frame 130, because two spheres are both measured in this frame, the fitting values are only related to the measurement accuracy of our FPP sensor, and are not affected by any sensor pose estimation error. Specifically, for each kind of data source, we manually cropped the corresponding points that belong to the two sphere surfaces, and fitted the diameter and standard deviation values using the *Geomagic* software.

Table 1 shows the comparison results of diameter and standard deviation fitting values of two spheres. The standard deviation values directly reflect the surface consistency of our reconstruction model. After the global optimization, it decreases from 0.1971 mm to 0.0282 mm for sphere 1, and decreases from 0.2534 mm to 0.0301 mm for sphere 2. Furthermore, the standard deviation value of globally-optimized model is very close to the value of a single measurement (frame 130), which demonstrates that our reconstructed surface exhibits very good shape consistency.

We also compared the difference of the sphere center distances between not-optimized and globally-optimized models, as shown in Table 2. The absolute error of sphere center distance relative to the ground truth decreases from 0.2080 mm to 0.0205 mm, the relative error relative to the ground truth decreases from 0.1387% to 0.0137%.

Both of the above two comparison results explain the surface shape inconsistency refinement from Figure 9a,b to Figure 10a,b, and illustrate that with the global optimization (1) the accumulated error is substantially reduced to less than 1/10 of the not-optimized reconstruction result, (2) the final sensor pose estimation accuracy can well match the measurement accuracy of our FPP sensor.

## 6. Conclusions

In this paper, we present a high-accuracy globally consistent surface reconstruction method using fringe projection profilometry. The accumulated sensor pose estimation error problem is solved with a first relative sensor pose estimation step and a following global sensor pose optimization step. The former step tries to reduce the accumulated error by maximizing the relative sensor pose estimation accuracy; it helps to ensure the initial sensor poses lie in the convergence basin of the following global optimization method. The latter step globally optimizes the sensor poses through a multi-view point cloud registration formulated in the pose graph optimization framework. Besides, adaptive keyframe selection and loop closure detection method are proposed to efficiently and automatically build point cloud connections and their relative pose constraints, which are the prerequisites of global sensor pose optimization. By qualitatively observing and quantitatively analyzing the reconstruction results of a 1300 mm × 400 mm workpiece, we validated the effectiveness and accuracy of our method. Our method demonstrates the ability to accomplish industrial-level surface model reconstruction without any external positional assistance but only using a single FPP sensor.

Since our reconstruction method is based on 3D registration, it also shares some limitations similar to most 3D registration based surface reconstruction methods [7,12,16]. For example, when the target object is near a plane, 3D registration may not converge to a correct result due to insufficient geometric constraint [11], which will stop the sensor poses from being robustly tracked. A possible solution is to further exploit the usage of surface textures constraint to help the robust tracking of sensor poses.

## Figures and Tables

**Table 1:** Comparison of the diameter and standard deviation fitting results between not-optimized and globally-optimized model.

	Data Source	Diameter (mm)	Standard Deviation (mm)
	not-optimized model	44.0074	0.1971
Sphere 1	globally-optimized model	**43.9713**	**0.0282**
	Frame 130	44.1121	0.0164
	not-optimized model	43.8685	0.2534
Sphere 2	globally-optimized model	**44.0624**	**0.0301**
	Frame 130	44.0881	0.0258

**Table 2:** Sphere center distance fitting results with the absolute and relative errors relative to the ground truth.

Data Source	Sphere Center Distance (mm)	Absolute Error (mm)	Relative Error (%)
not-optimized model	149.7950	0.2080	0.1387
globally-optimized model	**149.9825**	**0.0205**	**0.0137**
Frame 130	150.0030	/	/
